# Antimicrobial Properties of Encapsulated Antimicrobial Natural Plant Products for Ready-to-Eat Carrots

**DOI:** 10.3390/foods8110535

**Published:** 2019-11-01

**Authors:** Yosra Ben-Fadhel, Behnoush Maherani, Melinda Aragones, Monique Lacroix

**Affiliations:** Research Laboratories in Sciences Applied to Food, Canadian Irradiation Center, INRS–Armand Frappier, Health and Biotechnology Center, Institute of Nutraceutical and Functionals Foods, 531 Boulevard des Prairies, Laval, QC H7V 1B7, Canada; Yosra.bf@gmail.com (Y.B.-F.); bmaherani@gmail.com (B.M.); melindaragones@gmail.com (M.A.)

**Keywords:** natural antimicrobials, encapsulation, shelf-life, microbiological quality

## Abstract

The antimicrobial activity of natural antimicrobials (fruit extracts, essential oils and derivates), was assessed against six bacteria species (*E. coli* O157:H7, *L. monocytogenes*, *S*. Typhimurium, *B. subtilis*, *E. faecium* and *S. aureus*), two molds (*A. flavus* and *P. chrysogenum*) and a yeast (*C. albicans*) using disk diffusion method. Then, the antimicrobial compounds having high inhibitory capacity were evaluated for the determination of their minimum inhibitory, bactericidal and fungicidal concentration (MIC, MBC and MFC respectively). Total phenols and flavonoids content, radical scavenging activity and ferric reducing antioxidant power of selected compounds were also evaluated. Based on in vitro assays, five antimicrobial compounds were selected for their lowest effective concentration. Results showed that, most of these antimicrobial compounds had a high concentration of total phenols and flavonoids and a good anti-oxidant and anti-radical activity. In situ study showed that natural antimicrobials mix, applied on the carrot surface, reduced significantly the count of the initial mesophilic total flora (TMF), molds and yeasts and allowed an extension of the shelf-life of carrots by two days as compared to the control. However, the chemical treatment (mix of peroxyacetic acid and hydrogen peroxide) showed antifungal activity and a slight reduction of TMF.

## 1. Introduction

Plants, spices, fruits and vegetable extracts have been exploited since antiquity for their aromas, coloring ability, antioxidant and antimicrobial properties [1]. However, at the beginning of the 19th century, a rapid rise of the use of chemical additives has been observed. Among the chemical additives used in food, nitrites, sulfide dioxide, sulfites, parabens, peroxyacetic acid and hydrogen peroxide are the best known. However, these additives are controversial as many have shown potential health risks, mainly carcinogenic effects, irritation and the appearance of resistant strains [1,2]. There is, therefore, a growing interest in identifying natural antimicrobial extracts which have the advantage of being effective with much less toxic and less allergenic effects. Natural antimicrobial extracts have demonstrated various antiviral, antifungal, antibacterial, anti-parasitic, antioxidant, and even insecticidal activities [3,4]. For example, it was demonstrated that garlic juice and tea extract could inhibit bacteria even those resistant to antibiotics, such as ciprofloxacin, methicillin and vancomycin [5]. In addition to their antimicrobial properties, natural antimicrobials often have functional properties already used as anticancer, radioprotective and hypoglycemic [1]. For example, it was observed that lime juice extract can inhibit the growth of pancreatic cancer cells [6]. Antioxidant properties have also been reported for certain plant extracts like garlic and onion. Antioxidant properties can help in the prevention of meat discoloration, the preservation of vitamin content (B_1_ and B_2_) and the prevention of lipid oxidation [7]. Some of the active compounds present in plants, herbs, spices, fruits and vegetables are known as secondary metabolites. The main groups of compounds responsible for the antimicrobial activity of plants extracts include phenols (phenolic acids, flavonoids: i.e., flavonols, tannins), quinones, saponins, coumarins, terpenoids and alkaloids [8]. Natural extracts under the form of essential oils are rich in flavonoids, terpenes, terpenoids and aromatic and aliphatic constituents and could be obtained by hydro or steam distillation, solvent extraction, ultrasound, microwave, ohmic heating, supercritical CO_2_ extraction or pulsed electric field [3]. Most of their active compounds are found in leaf extract (i.e., rosemary, sage), flowers and flower buds (i.e., cloves), bulbs (i.e., garlic, onion), rhizomes (i.e., asafetida) and fruits (i.e., pepper) [9]. Depending on plant type and bacterial strain, essential oil derivatives could have a high antibacterial activity. Bertoli, et al. [10] reported that 60% of plant essential oils have antifungal activity. Their mode of action on microorganisms has been the object of several studies and demonstrated that essential oils, due to their hydrophobic nature, are able to react with the lipid layer of the bacterial cell membrane, thereby increasing the permeability of membranes inducing leakage of ions and cell contents, lysis and death of bacteria [11]. Their efficiency against several bacteria, molds and yeasts made of the essential oils a good candidate for food industry to insure food safety. Unfortunately, their use in food industry is restricted by a low dose due to their strong sensorial impact and toxicity [12,13]. On the other hand, the hydrophobic nature of essential oils affects their homogeneity and bioavailability on the food surface. Their encapsulation in a more suitable matrix could help to avoid this inconvenient and can prevent volatilization and oxidation of their active compounds. Moreover, encapsulation could mask the strong aroma and prevent the degradation of the active compounds [14].

Carrots have been implicated in several outbreaks in England and Wales during 1992–2005, in the United States during 1973–1997 [15,16] and in 2004 [17]. The most frequent pathogens involved in these outbreaks are *E. coli* O6 (strain that produced the heat-stable and heat labile toxins (O6: NM LT ST), VTEC, *Yersinia pseudotuberculosis* which caused gastrointestinal illness and erythema nodosum among schoolchildren in Finland and *Shigella sonnei* [15,18]. Others studies demonstrated the possibility of growth of *Salmonella spp*. and *Listeria monocytogenes* on carrots [19]. The fungal strains of *Alternaria, Rhizopus*, *Aspergillus, Stemphylium* and *Botrytis* were also found to contaminate carrots [20,21]. The mechanism of contamination of carrots remains not well known. Monaghan and Hutchison [22] reported inadequate hand hygiene in the field can transfer bacterial contamination to hand-harvested carrots. Direct contact with wildlife feces during storage and cross-contamination of the equipment during washing and peeling could also be contributing factors [16].

The main objective of this study was to assess the antimicrobial activities of 17 antimicrobial agents against nine different microorganisms (Gram negative, Gram positive, molds and yeast) that could affect food products in order to select the most efficient antimicrobial extracts. The total phenols and flavonoids content, the anti-radical and antioxidant activity were assessed for each selected extract. In this study, a strategy was developed in order to reduce the efficient dose of natural antimicrobial extracts by the development of formulation containing a mixture of natural extracts encapsulated in o/w emulsion which could act in synergy. Then, the antimicrobial efficiency of the antimicrobial-loaded emulsion was tested in situ onto pre-cut carrots. Finally, sensorial evaluation was done on the treated carrots.

## 2. Materials and Methods

### 2.1. Antimicrobial Extracts

Biosecur F440D (33–39%) was provided by Biosecur Lab, Inc. (Mont St-Hilaire, Québec, QC, Canada). Citral was provided from BSA, Inc. (BSA Ingredients s.e.c/l.p., Montreal, QC, Canada). Cranberry juice (*Vaccinium macrocarpon*) was provided by Atoka Cranberries, Inc. (Manseau, QC, Canada) and was stored at −80 °C until used. Fourteen essential oils from spices, fruits and plants were bought from Biolonreco, Inc. (Dorval, QC, Canada) and their main constituents are presented in Table 1. Biosecur F440D, citral and essential oils were stored at 4 °C.

### 2.2. Preparation of Bacterial Cultures

Six bacterial strains, four Gram positive: *Listeria monocytogenes* HPB 2812 (Health Canada, Health Products and Food Branch, Ottawa, Canada), *Staphylococcus aureus* ATCC 29213 (American Type Culture Collection, Rockville, MD, USA), *Enterococcus faecium* ATCC 19434 (American Type Culture Collection, Rockville, MD, USA) and *Bacillus subtilis* ATCC 23857 (INRS-Institut Armand-Frappier, Laval, QC, Canada), and two Gram negative: *Escherichia coli* O157:H7 (EDL 933, provided by Pr. Charles Dozois) and *Salmonella* Typhimurium SL 1344 (INRS-Institut Armand-Frappier, Laval, QC, Canada) were used as target bacteria in antimicrobial tests. *Aspergillus flavus* (INRS-Institut Armand-Frappier, Laval, QC, Canada) and *Penicillium chrysogenum* (INRS-Institut Armand-Frappier, Laval, QC, Canada) were used as fungal strains and *Candida albicans* ATCC10231 (INRS-Institut Armand-Frappier, Laval, QC, Canada) as yeast. All the bacteria were stored at −80 °C in Tryptic Soy Broth medium (TSB; BD, Franklin Lakes, NJ, USA) containing glycerol (20% *v*/*v*). Before each experiment, bacterial stock cultures were propagated through two consecutives 24 h growth cycles in TSB at 37 °C to reach the concentration of approximately 10^9^ CFU/mL. The grown cultures were then diluted in sterile peptone water 0.1% (Alpha Biosciences, Inc., Baltimore, MD, USA) to obtain a working culture of approximately 10^6^ CFU/mL.

For fungal evaluation, *A. flavus and P. chrysogenum* were propagated through 72 h growth cycle on potato dextrose agar (PDA, Difco, Becton Dickinson) at 28 ± 2 °C. Colonies were isolated from the agar media using sterile platinum loop, suspended in sterile peptone water, and filtrated through sterile cell strainer (Fisher scientific, Ottawa, ON, Canada). *C. albicans* was inoculated in potato dextrose broth (PDB, Difco, Becton Dickinson) for 24 h at 28 °C. The filtrate was adjusted to 10^6^ CFU/mL using a microscope before dilution to reach approximately 10^6^ CFU/mL for the disk diffusion agar and the minimum inhibitory, bactericidal and fungicidal concentration (MIC, MBC and MFC, respectively) determination [23].

### 2.3. Preliminary Study

First, 100 µL of the tested microorganisms 10^6^ CFU/mL were seeded on sterile Petri dishes containing Muller Hinton Agar (MHA, BD, Franklin Lakes, NJ, USA). Then, 5 µL of each pure antimicrobial compounds were deposited on the surface of a sterile 6-mm filter disk. A negative control was used by deposing 5 µL of sterile water on the surface of the disk. All plates were sealed with parafilm to avoid evaporation and incubated for 24 h at 37 °C for bacteria and for 48 h to 72 h at 28 °C for molds and yeasts followed by the measurement of the diameter zone of the inhibition expressed in mm. On the basis of the disk diffusion results, the most efficient antimicrobial compounds have been selected to determine their MIC, MBC and MFC, their total phenols and flavonoids content and their antioxidant and anti-radical properties and to evaluate the in situ antimicrobial efficiency of the mixture on pre-cut carrot surface.

### 2.4. Antimicrobial Efficiency

The minimum inhibitory concentration (MIC) and the minimum bactericidal and fungicidal concentration (MBC and MFC) were determined on the emulsion as an encapsulation form composed of essential oils 2.5% (*w*/*w*), tween 80 2.5% (*w*/*w*) and 95% (*w*/*w*) distilled water. The mixture was homogenized by vortex for 1 min and by Ultra-Turrax (IKA T25 digital Ultra-Turrax disperser, IKA Works Inc., Wilmington, NC, USA) for 1 min at 15,000 rpm. Because of its water solubility, Biosecur F440D was prepared at 0.4% (*w*/*w*) in distilled water. All the prepared solutions were then filtered through 0.2 µm syringe filter.

The MIC value of each antimicrobial compound was determined in sterilized flat-bottomed 96-well microplate according to the serial microdilution method [23]. Briefly, serial dilutions (200:100 μL) of the antimicrobial compounds were made in Mueller Hinton Broth (MHB, Difco, Becton Dickinson) for bacteria and in Potato Dextrose Broth (PDB, Difco, Becton Dickinson) for molds and yeast and dispensed into 96-well microplates to obtain a dilutions range of 2000–15 ppm for Biosecur F440D and 12,500–145 ppm for essential oils. Then, a volume of 15 µL of bacteria, molds and yeast suspension (10^6^ CFU/mL) was added. Two control samples were evaluated; the 1st was to control the growth of the evaluated microorganisms where a volume of 100 µL of MHB/PDB was mixed to 15 µL of the selected microorganism. The 2nd control was the blank where a volume of 15 µL of distilled water was added to 100 µL of each antimicrobial dilution. The MIC of tween 80 at 2.5% was also evaluated. The final volume in all the wells was 115 µL. Microplates were sealed with acetate foil to avoid evaporation and then incubated on a shaker (Forma Scientific. Inc., Marietta, OH, USA) at 80 rpm at 37 °C for 24 h and 28 °C for 48 h respectively for bacteria and molds/yeasts to insure a better homogenization. The absorbance was then measured at 595 nm in an absorbance microplate reader (BioTek ELx800^®^, BioTek Instruments Inc., Winooski, VT, USA). The MIC is considered to be the lowest concentration of the antimicrobial compounds that completely inhibits bacterial and fungal strain growth by showing equal absorbance as blank. Afterwards, to assess the MBC and the MFC, 5 µL of each well were taken from the microplate and were deposit on a Petri dish containing Tryptic Soy Agar (TSA) for bacteria and PDA for molds and yeasts. Finally, Petri dishes were incubated for 24 h at 37 °C for bacteria or 48–72 h at 28 °C for molds and yeasts respectively. The MBC and the MFC were respectively determined as the concentration where no colony was detected.

### 2.5. Total Phenol Determination

The total phenol content was carried out using a Folin–Ciocalteu colorimetric method according to Dewanto, et al. [24]. Pure essential oils and Biosecur F440D were diluted in anhydrous ethanol and water respectively to obtain suitable dilution within the standard curve ranges of 0–200 µg of gallic acid/mL. Measurements were done at 760 nm versus the blank prepared similarly with water or ethanol. All values were expressed as mean (milligrams of gallic acid equivalents per g of antimicrobial compounds).

### 2.6. Radical Scavenging Activity (DPPH)

The antioxidant activity of the antimicrobial compounds was determined using 2,2-diphenyl-1-picrylhydrazyl (DPPH) as a free radical [25]. The reaction for scavenging DPPH radicals was performed in polypropylene tubes at room temperature. One milliliter of a 40 µM of methanolic solution of DPPH was added to 25 µL of diluted antimicrobial compounds. The mixture was shaken vigorously and left for 90 min. The absorbance of the resulting solution was measured at 517 nm. Anhydrous methanol was used as a blank solution, and DPPH solution without any sample served as control. The Trolox equivalent antioxidant capacity (TEAC) values were calculated from the equation determined from linear regression after plotting known solutions of Trolox or ascorbic acid with different concentrations (0–1 mM). The DPPH inhibition percentage was calculated using Equation (1) and the antiradical activity was expressed as mM of Trolox or ascorbic acid.

Radical scavenging activity (%) = (Control OD − Sample OD) × 100/Control OD (1)

### 2.7. Ferric-Reducing Antioxidant Power (FRAP)

Total antioxidant activity was estimated by FRAP assays [26]. Three aqueous stock solutions containing 0.1 M acetate buffer (pH 3.6), 10 mM TPTZ [2,4,6-tris(2-pyridyl)-1,3,5-triazine] in 40 mM hydrochloric acid solution, and 20 mM ferric chloride were prepared and stored under dark conditions at 4 °C. Stock solutions were combined (10:1:1, *v*/*v*/*v*) to form the FRAP reagent just prior to analysis. FRAP reagent was heated in a water bath for 30 min at 37–40 °C. For each assay, 2.8 mL of FRAP reagent and 200 µL of diluted sample were mixed. After 10 min, the absorbance of the reaction mixture was determined at 593 nm. The standard curve was prepared with ascorbic acid (0–2 mM). Results were expressed as equivalent µM of ascorbic acid per gram of antimicrobial.

### 2.8. Determination of Total Flavonoids Content

Total flavonoids content was determined by using a colorimetric method [24]. Briefly, 0.25 mL of diluted antimicrobial compounds or (+) catechin standard solution was mixed with 1.25 mL of distilled water followed by the addition of 75 µL of a 5% NaNO_2_ solution. After 6 min, 150 µL of a 10% AlCl_3_ 6H_2_O solution was added and allowed to stand for 5 min at room temperature before 0.5 mL of 1 M NaOH was added. The mixture was brought to 2.5 mL with distilled water and mixed well. The absorbance was measured immediately against the blank at 510 nm in comparison with the standards prepared similarly with known (+)-catechin concentrations. The results were expressed as mean (micrograms of catechin equivalents per gram of antimicrobial).

### 2.9. In Situ Test on Pre-Cut Carrots

#### 2.9.1. Antimicrobial Loaded Emulsion

To encapsulate the natural antimicrobial compounds, an emulsion was prepared by mixing Biosecur F440D^®^ to citrus, Asian, Mediterranean and pan tropically essential oils composed mainly with lemongrass, oregano and cinnamon essential oils respectively [27]. Sunflower lecithin (HLB 7) and sucrose monopalmitate (HLB 18) were used as emulsifiers (180 ppm) to obtain a stable emulsion with a HLB = 12 and an oil phase: emulsifier’s ratio of 1:1. The emulsion was magnetically homogenized then mixed with Ultra-Turrax at 10,000 rpm for 1 min.

#### 2.9.2. Samples Preparation

Freeze pre-cut carrots were provided by Bonduelle, Inc. (Sainte-Martine, Canada). Carrot was washed with water then divided into 3 groups: untreated carrots (control), treated carrots with antimicrobial formulation-loaded emulsion (containing a mixture of Biosecur F440D extract and Asian, Mediterranean, citrus and pan tropical essential oils) and treated carrots with commercial chemical antimicrobial (0.03% of Tsunami: a mix of 15.2% of peroxyacetic acid and 11.2% of hydrogen peroxide). For treated samples, carrots were dipped in the antimicrobial solution for 30 s, kept drying under laminar flow hood for 15 min to discard the exceeding solution. Samples were then stored in Whirl-Pak™ Sterile Filter Bags (Nasco, Whilpack^®^, Fort Atkinson, WI, USA) at 4 °C for 8 days (20 g per bag). Emulsifiers were considered too low to not affect the antimicrobial activity of the emulsion.

#### 2.9.3. Shelf-life Estimation

The total mesophilic bacterial count (TMF) was evaluated during 8 days of storage at 4 °C. The TMF was selected based on previous studies, as TMF contains a complex mix of different autochthonous microorganisms including *Candida* spp. [28], *Entrobacter* spp., *Salmonella* spp. and *S. aureus* [29]. To estimate the initial count of TMF, a bacterial analysis was carried out for the control on day 0. During storage, all treatments and control were evaluated on day 1, 3, 6 and 8. On each day of analysis, 60 g of 0.1% (*w*/*v*) peptone water (Alpha Biosciences Inc., Baltimore, MD, USA) were added to filter bag containing 20 g of carrots previously prepared. The carrot samples were mixed during 2 min at high speed (260 rpm) in a Lab-blender 400 stomacher (Laboratory Equipment, London, UK), then 100 µL were seeded on TSA for TMF evaluation and on PDA with chloramphenicol for molds and yeasts evaluation. Plates were incubated at 37 °C and 28 °C during 48–72 h for TMF and molds and yeast respectively. Results were expressed as bacterial count and fungal count (log CFU/g) during storage at 4 °C.

Shelf-life limit was considered at the limit of unacceptability, when TMF count and the total molds and yeasts reached the current authorities regulation level of 10^7^ CFU/g and 10^4^ CFU/g, respectively [30]. Equation (2) was used to describe the growth of bacteria (*Y*) over time during the exponential phase.

*Y* = *X*exp (*μt*) (2)

where *X* is the initial population, *μ* the growth rate of TMF (Ln CFU/g/Day) and *t* the number of storage days.

### 2.10. Sensory Evaluation

In order to evaluate the effect of the developed antimicrobial formulation on the sensory properties of carrots, the sensory evaluation, was carried out by comparing the control to treated carrots with the developed antimicrobial formulation. The sensorial evaluation of treated and untreated carrots was done using a hedonic test [31]. The level of appreciation was determined using nine points (1 = dislike extremely; 5 = neither like nor dislike; 9 = like extremely). Samples were treated with the antimicrobial formulation-loaded emulsion (containing a mixture of Biosecur F440D and Asian, Mediterranean, citrus and pan tropical essential oils) and kept to dry. The sensorial evaluation was done by a panel of 24 untrained people after 1 day of the treatment application. For each panelist, 3 pieces of carrots were served to evaluate the flavor, the odor and the global appreciation. Treated samples consisted of carrot samples coated with the antimicrobial formulation.

### 2.11. Statistical Analysis

Each experiment was done in triplicate (*n* = 3). For each replicate 2 samples were analyzed. Analysis of variance (ANOVA), Duncan’s multiple range tests for equal variances and Tamhane’s test for unequal variances were performed for statistical analysis using SPSS 18.0 software (SPSS Inc., Chicago, IL, USA). Differences between means were considered significant when the confidence interval was lower than 5% (*p* ≤ 0.05).

## 3. Results

### 3.1. Preliminary Study

Results of the disk diffusion method (Table 2) showed that from 17 evaluated antimicrobial compounds, five antimicrobial agents that showed high inhibitory diameter against all the tested microorganisms were identified. Based on their bioactivity, these antimicrobial compounds could be also grouped into four distinctive groups: Group 1 contains pan tropical, Mediterranean and thyme essential oils which have a large spectral activity against bacteria, yeast and molds with an inhibitory diameter ≥23.7 mm. Their effectiveness was higher against yeast and molds with an inhibitory diameter between 38.3 and 80 mm for *C. albicans*, *P. chrysogenum*, and *A. flavus* as compared to an inhibitory diameter between 23.7 and 44.3 mm for *S*. Typhimurium, *L. monocytogenes*, *B. subtilis*, *E. coli*, *S. aureus* and *E. faecium*. Group 2 contains Asian, cloves, citrus and thyme savory leaves essential oils and citral and was very efficient to inhibit molds and yeasts. Asian essential oil and citral showed an average antibacterial activity against six bacterial strains with an inhibitory diameter ≤22.5 mm and an antifungal activity with an inhibitory diameter between 23.0 mm and 80.0 mm. Citrus and cloves essential oils were efficient to reduce *B. subtilis*, *S. aureus, C. albicans, A. flavus* and *P. chrysogenum* showing an inhibitory diameter between 22.0 and 68.7 mm. Otherwise, they showed above-average efficiency against the other microorganisms. Group 3 contains Biosecur F440D which possesses a good antimicrobial activity against all the microorganisms. The inhibitory diameter of Biosecur F440D varied from 12.3 mm to 25.4 mm for *E. faecium* and *S. aureus*, respectively, showing a medium antimicrobial activity whether against bacteria molds or yeast. Biosecur F440D was more efficient to inhibit bacteria, molds and yeasts than cranberry juice. Group 4 contains bergamot, marjoram, peppermint, sweet orange, tea tree, myrtle and ginger essential oils and cranberry juice, and showed a very low antimicrobial activity. Pepper mint essential oil was efficient only to inhibit the growth of *C. albicans* showing an inhibitory diameter of 31.3 mm. Results showed that essential oils of bergamot, sweet marjoram, sweet orange, myrtle and ginger with an inhibitory diameter ≤18.3 mm showed a very low antimicrobial activity against bacteria, molds and yeasts.

Based on these results, five antimicrobial extracts were selected to characterize their MIC, MBC, MFC and to determine their total phenols and flavonoids composition and their antiradical and antioxidant properties: citrus and Asian essential oils for their antifungal activity, pan tropical and Mediterranean essential oils for their large spectral activity and Biosecur F440D for its good activity and its hydrophilic properties.

### 3.2. Determination of MIC, MBC and MFC

The results of MIC, MBC and MFC of the selected antimicrobial compounds are presented in Table 3. Results showed that Biosecur F440D was the most efficient in inhibiting the bacterial growth, showing a MIC and a MBC between 17 and 171 ppm against all evaluated bacterial strains. Pan tropical essential oil was also more efficient in inhibiting the growth of molds and *C. albicans* showing a fungicidal activity against *A. flavus* and *P. chrysogenum* with a MFC between 155 and 621 ppm. Pan tropical and Mediterranean essential oils showed the highest antimicrobial activity against almost all microorganisms tested showing a bactericidal and fungicidal activity. They inhibited the growth of all evaluated microorganisms at a concentration ≤1241 ppm for pan tropical essential oil and ≤2474 ppm for Mediterranean essential oil. These results indicate that these two essential oils have an interesting antimicrobial potential. Asian essential oil showed a high activity in inhibiting the growth of molds and yeast and showed a MFC of 311, 622 and 4979 ppm for *C. albicans*, *A. flavus* and *P. chrysogenum,* respectively. On the other hand, Biosecur F440D had a bactericidal activity against all the evaluated bacterial strains as compared to essential oils which have fungicidal activity.

### 3.3. Total Phenols and Flavonoids

Results of total phenols and total flavonoids content (Table 4) showed that Mediterranean and pan tropical essential oils were highly concentrated in total phenols (respectively 220.57 and 34.62 mg gallic acid equivalent/ g of antimicrobial) and total flavonoids (respectively 34.62 and 17.63 mg catechin equivalent/g of antimicrobial). Biosecur F440D showed a concentration of 4.38 mg gallic acid equivalent/g of antimicrobial for total phenols content and 1.26 mg catechin equivalent/g of antimicrobial for total flavonoids. Citrus and Asian essential oils showed the least concentration of total phenol and flavonoid content with, respectively, 1.51 and 1.41 mg gallic acid equivalent/g of antimicrobial and 0.06 and 0.56 mg catechin equivalent/g of antimicrobial.

### 3.4. Radical Scavenging Activity and FRAP

Biosecur F440D and Mediterranean, Asian, pan tropical and citrus essential oils were tested for their ability to scavenge radicals by the DPPH method. Biosecur F440D has the highest radical scavenging activity above all the other compounds with 0.28 mM of Trolox (Table 5). The radical scavenging of Biosecur F440D was two times higher than Mediterranean essential oil (0.18 mM equivalent), three times higher than citrus essential oil (0.07 mM equivalent) and 10 times higher than Asian essential oil (0.02 mM of Trolox equivalent).

The antioxidant activity measured with the ferric reducing power assay revealed similar results to those obtained with the DPPH technique (Table 5). The highest antioxidant activities were obtained with Mediterranean essential oil (0.76 Eq µM of ascorbic acid equivalent/g of extract), followed by pan tropical essential oil and Biosecur F440D (0.43 and 0.30 Eq µM of ascorbic acid equivalent/g of antimicrobial respectively). Asian and citrus essential oils have the lowest values (below 0.04 Eq µM of ascorbic acid equivalent/g of antimicrobial).

### 3.5. In Situ Analysis

**Figure 1:**
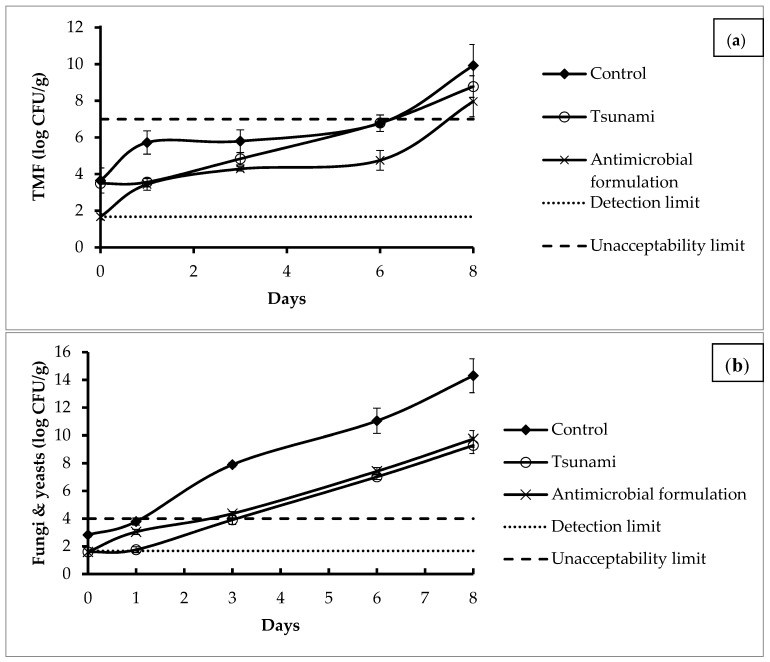
Total mesophilic flora (**a**) and total molds and yeasts (**b**) growth on pre-cut carrots.

Results of the growth of TMF, molds and yeasts (Figure 1) showed that on Day 0, the encapsulation of the antimicrobial formulation in o/w emulsion (containing a mixture of Biosecur F440D and Asian, Mediterranean, citrus and pan tropical essential oils), applied on the surface of carrots, allowed 2 log reductions for TMF and 1 log reduction for molds and yeasts as compared to the control (*p* ≤ 0.05). The mix of selected antimicrobial ingredients-loaded emulsion was more effective than the commercial mix (Tsunami 100). A significant reduction of TMF, molds and yeasts counts was also observed during the whole storage period showing a 1 log reduction of TMF on carrots treated with the antimicrobial ingredients-loaded emulsion as compared to the control which signifies a better control of the microbiological growth of TMF on pre-cut carrots. The antimicrobial activity of the commercial mix of peroxyacetic acid and hydrogen peroxide against TMF was also lower than the antimicrobial ingredients-loaded emulsion during the whole storage. The shelf-life of pre-cut carrots was reached on Day 6 for untreated carrots, treated carrots with the commercial chemical preservatives and on Day 8 for treated carrots with the developed antimicrobial-loaded emulsion (Figure 1a). By considering Days 1, 3 and 6, the growth rate was also lower in treated carrot with the antimicrobial formulation and with Tsunami samples showing a growth rate of 0.1291 and 0.1852 Ln CFU/g/day respectively as compared to 0.2193 Ln CFU/g/day for untreated samples (Table 6).

By considering the results of total molds and yeasts (Figure 1b), the shelf-life of pre-cut carrots was reached on Day 1 for untreated carrots and on Day 3 for both treated carrots with the antimicrobial-loaded emulsion and treated carrots with the chemical preservative (Tsunami). The obtained in situ results indicated that the antimicrobial formulation was effective against TMF and molds and yeasts, not only immediately after treatment but also during a mid-term storage.

### 3.6. Sensory Evaluation

**Figure 2:**
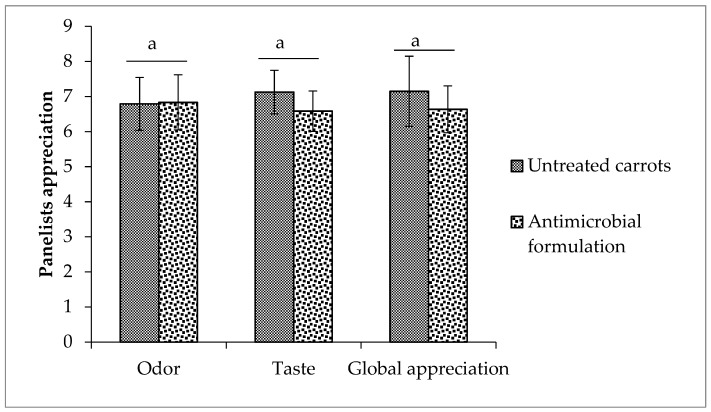
Effect of antimicrobial treatment on sensorial properties of pre-cut carrots.

Sensory analysis of pre-cut carrots treated or not with the antimicrobial formulation-loaded emulsion, was done by evaluating its odor, taste and global appreciation, using a nine-point hedonic scale and a panel of 24 untrained people and results are presented in Figure 2. Results showed that the antimicrobial treatment did not have any detrimental effect on the sensorial quality of the coated carrots. The values of the odor, the taste and the global appreciation were 6.8, 6.6 and 6.6 for the carrots treated with the antimicrobial formulation as compared to 6.8, 7.1 and 7.2 for the control samples. The odor was not affected by the applied treatment and a slight reduction on the attributed note was observed on the taste and the global appreciation. Overall, no significant negative effect (*p* > 0.05) was observed.

## 4. Discussion

Valorization of natural antimicrobials has been extensively investigated during the last decades. In the present study, it was demonstrated that natural antimicrobials have a good antioxidant and antimicrobial activity against a wide range of food pathogens and spoilage microorganisms, and that their combination allows a better control of the microbiological quality of pre-cut carrots without altering their sensory properties.

Using the disk diffusion method, we have identified five antimicrobial compounds that showed a high inhibitory diameter against the tested microorganisms: Biosecur F440D and citrus, Asian, Mediterranean and pan tropical essential oils. Similar results for inhibitory diameter obtained by disk diffusion were also reported by Baser and Buchbauer [13] for cinnamon and citronella against *L. monocytogenes* and *S*. Typhimurium. Despite the medium inhibitory diameter (12.5–25.4 mm) of Biosecur F440D as compared to essential oils, its MIC and MBC was the lowest against all the evaluated bacteria. According to Ghabraie, et al. [32] and Lopez, et al. [33], the antimicrobial activity of essential oils is due to both solid and vapor-phase fractions. The antimicrobial activity of the vapor-phase could be observed only when essential oils are seeded on surface which was the case with the disk diffusion method. With the MIC method, the antimicrobial evaluation was done in liquid medium which reduces significantly the antimicrobial effect of the vapor fraction. However, Biosecur F440D, because of its water solubility, has a bactericidal activity when employed in liquid media and the obtained MIC was similar to the MBC (Table 3). Results obtained with disk diffusion agar confirmed previous observations and showed a higher or similar sensitivity of Gram positive bacteria to essential oils than Gram negative [13,34]. On the other hand, results obtained with MIC and MBC of essential oils showed that overall, essential oils were more efficient to inhibit Gram-negative bacteria than Gram positive as well showing a lowest MIC and MBC. These results suggest that volatile compounds in essential oils (MW < 300) could have a higher efficiency against Gram negative probably due to its various chemical compounds: alcohols, ethers or oxides, aldehydes, ketones, esters, amines, amides, phenols, heterocycles, and mainly the terpenes. It is known that the composition has an impact on the antimicrobial efficiency [35].

The antimicrobial behavior observed in the *in vitro* study of each antimicrobial compound differs mainly due to the difference in their chemical composition and nature. The Mediterranean and the pan tropical essential oils are highly effective antimicrobial compounds, leads to a significant inhibition against almost all evaluated microorganisms.

The Mediterranean essential oil, for example, is rich in total phenols and total flavonoids (Table 4). Similar results were observed by Wogiatzi, et al. [36] where several oregano origins were evaluated. Wogiatzi, Gougoulias, Papachatzis, Vagelas and Chouliaras [36] demonstrated that the total phenol content is also intimately related to the plant area of cultivation (foot/middle mountain). The hydroxyl group (-OH) of the phenolic compounds could interact with the membrane cell of bacteria and reduce the pH gradient through the cytoplasmic membrane which disrupts its structure and causes the loss of intracellular ATP and cell death [37]. The -OH group can also bind to the active site of enzymes (i.e., ATPase, histidine carboxylase), thereby altering the cellular metabolism of microorganisms [37,38]. The presence of phenolic compounds is also responsible for the good antioxidant activity of the Mediterranean essential oil observed, which act as free radical terminators [39]. Mediterranean essential oil is thus able to reduce the redox potential of the culture medium and to reduce the growth of microorganisms.

The antimicrobial activity of pan tropical essential oil is related to its high concentration on cinnamaldehyde. Cinnamaldehyde is capable of modifying the lipid profile of the microbial cell membrane probably due to its high antioxidant activity [40] which allows it to oxidase lipids on the bacterial membrane. Cinnamaldehyde can also inhibit the respiratory tract in certain bacteria by disrupting K^+^ and pH homeostasis [38]. In this study, pan tropical essential oil was also characterized by a great antifungal activity probably due to its ability to inhibit b-(1,3)-glucan and chitin synthesis in yeasts and molds which are the major structural compounds of the fungal cell walls [41].

Asian essential oil is highly concentrated on geranial and neral. These two isomers are the main compounds of the monoterpene citral which its antimicrobial activity is well known against several bacteria and molds [42,43]. Despite the antifungal effectiveness of Asian and citrus essential oils with disk diffusion method, the effectiveness in broth media was lower due probably to the ability of some microorganisms to transform citronellal and citral and other of their components to the sole carbon and energy source [13]. The antifungal activity of citral and cinnamaldehyde is the result of perturbation in ergosterol biosynthesis which causes a damage to the intracellular structure, loss of intracellular substance and membrane damage [44].

Citrus essential oil is highly concentrated with citronellol and geraniol, and showed a lower antimicrobial activity when compared to the other antimicrobials mainly due to the presence of only one double bond on its main compounds [37]. Nakahara, et al. [45] showed that citronellal and linalool has antifungal activity at a dose of 112 ppm. The antifungal activity of components found in citrus essential oil (i.e. mono-terpenes) was previously reported to the interference of such compounds with enzymatic reaction of wall, i.e., structure [46,47]. This allows a lack of cytoplasm, damage of integrity and finally the mycelial death [48]. Simic, et al. [49] showed also that the antimicrobial activity of citronella essential oil is intimately related to the association of citronella and citronellol due probably to a synergistic effect of their combination.

Biosecur F440D was efficient to inhibit the growth of Gram positive and Gram-negative bacteria showing a bactericidal activity. According to Álvarez-Ordóñez, et al. [50], citrus extracts at higher concentrations than the MIC, pore formation in the cell membrane is observed inducing leakage of nucleic acids. According to the same authors, to achieve a significant bacterial reduction, the exposure time or the antimicrobial concentration used should be two to four times higher than the MIC. Citrus extract mainly acts on the membrane. It causes conformational damage and/or compositional in some or all components of the cell membrane. It mainly affects the carboxyl groups of membrane fatty acids and thus impairs the macromolecular structure of the bacterial membrane. Several studies have tried to identify the components that are involved in the antimicrobial activity of citrus extract. It possesses strong antioxidant and antimicrobial properties, pleasant aromas and flavors, especially due to the presence of flavonoids. Citrus flavanones include naringenin, hesperidin, hesperitin and prunine and have a broad spectrum of action against many Gram-negative bacteria.

Citrus flavonoids have also a direct role in scavenging reactive oxygen species (ROS) as confirmed by the obtained results of antiradical activity [51]. This suggests that the ROS could be involved in the bactericidal activity observed on citrus extracts. Inoue, et al. [52] supported this suggestion and showed that ROS act in conjunction to induce the strong bactericidal activity. The antiradical activity is also due to the presence of vitamin C at a high concentration in citrus extract which is a natural free radical scavenger.

The obtained results of in vitro study showed a very good antimicrobial and antioxidant properties of the selected natural antimicrobials. As their mode of action against bacteria fungi and yeasts differs, the mix of natural antimicrobial-loaded emulsion applied on carrots as a food model, presented a large spectral activity against targeted microorganisms.

The application of this developed formulation encapsulated in o/w emulsion at a concentration that did not affect the sensory properties of carrots (Figure 2) was efficient to reduce TMF, molds and yeasts growth during storage at 4 °C. The developed formulation was also more effective than the chemical antimicrobial (mix of peroxyacetic acid and hydrogen peroxide) to control TMF and had similar efficiency to control molds and yeasts. Based on previous studies, the developed formulation seems to be also more effective than other chemical methods such as HOCl, 4% H_2_O_2_ which showed less than 2 log reduction of TMF of carrots [53]. In situ efficiency is mainly due to combined activity of different compounds. The use of such combination could help to better control spoilage of fruits and vegetables. According to Bassolé and Juliani [54], combining cinnamon and oregano yielded in most cases, in a synergistic activity against *E. coli* and *S*. Typhimurium. Monoterpene hydrocarbon (α-pinene) when mixed with limonene or linalool also showed additive and synergistic effects [54]. The obtained results present a new antimicrobial formulation based on natural plant extracts that allowed a better control of initial microflora that could replace the methods presently used in industries such as blanching and ozonized water.

## 5. Conclusions

This study showed that natural antimicrobial extracts are rich on antioxidant and antiradical compounds. Biosecur F440D has the highest radical scavenging activity and has a bactericidal activity against all evaluated bacteria. Pan tropical essential oil has particularly an antifungal activity. Mediterranean essential oil was highly rich on total phenol and has the highest antioxidant activity. The mixture of natural antibacterial extracts when encapsulated in o/w emulsion and applied on carrot surface showed a better antimicrobial effectiveness than commercial chemical treatment widely used to treat vegetables. The mixture could be used as food treatment to extend the shelf-life of pre-cut carrots by two days without affecting their sensory properties. Finally, this user-friendly antimicrobial formulation-loaded emulsion could be applied in the food industry as a way to fulfill federal regulation requirements.

## Figures and Tables

**Table 1:** List of organic essential oils (EO) and their composition.

Common Name	Botanic Name	Part	Compositions (%) *
Bergamote EO	*Citrus bergamia*	Zest	Limonene (36.2), Linalyle acetate (29.7), linalool (13.2), γ-terpinene (6.8), β-pinene (5.4)
Pan tropical EO	*Cinnamomum verrum*	Peel	E-cinnamaldehyde (55.1), cinnamyl acetate (9.6), β-caryophyllene (4.0)
Citrus EO	*Cymbopogon winterianus*	Aerial part	Citronellal (35.4), geraniol (20.1), Citronellol (12.2), elemol (4.6), Limonene (3.0), citronellyl acetate (2.9), germacrene D (2.7), geranyl acetate de (2.5), linalool (0.6)
Ginger EO	*Zingiber officinalis*	Rhizome	α-zingiberene (25.4), β-sesquiphellandrene + α-curcumene (13.9), Camphene (10.5), β-phellandrene + 1, 8-cineole (8.3), β-bisabolene + β-selinene (7.7), E,E-α-farnesene (4.2), α-pinene (3.3)
Asian EO	*Cymbopogon flexuosus*	Herb	Geranial (39.1), neral (31.6), geraniol (6.7), geranyl acetate (3.7)
Marjolaine shells EO	*Origanum majorana*	Flower top	Terpinene-4-ol (28.0), γ-terpinene (15.5), α-terpinene (9.5), Cis-thuyanol (7.3), α-terpineol (3.7)
Peppermint EO	*Mentha x piperita*	Aerial part	Menthol (30.6), menthone (29.3), 1,8-cineole + β-phellandrene (5.2), menthyl acetate (4.5), neomenthol (3.1), Isomenthone (4.4), menthofurane (4.2), Limonene (2.4)
Myrte cineole EO	*Myrtus communis*	leaf	α-pinene (51.5), 1,8-cineole (23.9), Limonene (10.4), Linalool (3.0)
Sweet orange EO	*Citrus sinensis*	Zest	Limonene (94.8)
Tea tree EO	*Melaleuca alternifolia*	Leaf	Terpinene-4-ol (37.6), γ-terpinene (21.1), α-terpinene (10.1), Terpinolene (4.8), 1,8-cineole + β-phellandrene (4.2), α-pinene (2.6), α-terpineol (2.5)
Mediterranean EO	*Origanum compactum*	Flower top	Carvacrol (46.1), thymol (17.6), γ-terpinene+ trans-β-ocimene (14.8), p-cymene (8.5)
Thyme leaf savory EO	*Thymus satureioides*	Flower top	Borneol (27.0), α-terpineol (11.9), camphene (10.5), α-pinene + α-thuyene (6.5), β-caryophyllene (5.5), Carvacrol (5.3), p-cymene (3.9), Linalol (3.7), Terpinene-4-ol + methyl carvacrol ether (2.9), 1,8-cineole + β-phellandrene (2.9), Thymol (2.8)
Cloves EO	*Eugenia caryophyllus*	Floral button	Eugenol (81.8), Eugenyl acetate (12.9), β-caryophyllene (3.4)
Thyme thymol EO	*Thymus vulgaris CT6*	Flower top	Thymol (46.6), p-cymene (16.9), γ-terpinene (9.3), Linalool (4.1), Carvacrol (3.5)

* Composition was provided by Biolonreco, Inc. and was determined by CPG-SM Hewlett Packard /CPG- FID; Column: HP Innowax 60-0.5-0.25; Carrier gas Helium: 22 psi.

**Table 2:** Inhibitory diameter of antimicrobials extracts against tested microorganisms (*n* = 3).

		Inhibition Diameter: Mean ± std.dev (mm)								
		Gram Positive				Gram Negative		Yeast	Molds	
		*L. monocytogenes*	*B. subtilis*	*E. faecium*	*S. aureus*	*S*. Typhimurium	*E. coli*	*C. albicans*	*A. flavus*	*P. chrysogenum*
**1**	**Biosecur F440D**	16.6 ± 1.9	18.9 ± 1.0	12.3 ± 0.7	25.4 ± 2.4	12.5 ± 1.1	13.7 ± 0.9	22.8 ± 1.6	14.1 ± 0.7	13.6 ± 3.0
**2**	**Cranberry juice**	8.7 ± 0.9	9.3 ± 1.6	6.0 ± 0.0	6.0 ± 0.0	6.0 ± 0.0	7.0 ± 1.2	6.0 ± 0.0	6.0 ± 0.0	6.0 ± 0.0
**3**	**Bergamote EO**	6.0 ± 0.0	13.7 ± 1.2	6.0 ± 0.0	6.0 ± 0.0	6.0 ± 0.0	6.0 ± 0.0	10.7 ± 0.4	6.0 ± 0.0	8.5 ± 0.3
**4**	Citrus EO ^*^	8.4 ± 0.6	68.7 ± 4.9	13.9 ± 0.4	35.6 ± 2.8	13.8 ± 1.0	14.8 ± 2.0	36.2 ± 5.5	22.4 ± 5.2	45.7 ± 4.0
**5**	**Cloves EO**	14.8 ± 1.2	24.9 ± 3.4	19.0 ± 1.9	22.0 ± 2.5	20.0 ± 0.8	20.1 ± 3.3	27.2 ± 0.7	38.6 ± 1.2	41.7 ± 1.2
**6**	**Marjoram EO**	13.9 ± 0.9	17.4 ± 3.5	16.1 ± 0.7	17.1 ± 0.8	17.3 ± 1.1	19.1 ± 2.4	13.0 ± 0.2	6.0 ± 0.0	11.2 ± 0.4
**7**	**Pepper menthe EO**	7.8 ± 0.4	19.1 ± 3.4	15.5 ± 0.9	18.9 ± 3.3	13.3 ± 0.4	14.4 ± 1.9	31.3 ± 1.8	6.0 ± 0.0	9.9 ± 1.1
**8**	**Sweet orange EO**	6.0 ± 0.0	14.0 ± 1.5	6.0 ± 0.0	6.0 ± 0.0	6.0 ± 0.0	6.0 ± 0.0	11.1 ± 0.9	6.0 ± 0.0	8.5 ± 0.1
**9**	**Mediterranean EO**	23.8 ± 0.5	44.3 ± 4.1	33.9 ± 4.4	42.7 ± 4.0	28.5 ± 3.6	27.2 ± 2.5	52.0 ± 1.6	59.0 ± 2.6	80.0 ± 0.0
**10**	**Tea tree EO**	12.2 ± 0.4	17.2 ± 1.6	16.5 ± 0.8	18.3 ± 3.9	16.7 ± 2.2	17.3 ± 1.7	12.3 ± 1.3	6.0 ± 0.0	9.5 ± 0.4
**11**	**Thyme savory leaves EO**	11.3 ± 0.3	21.3 ± 3.7	16.0 ± 0.9	27.6 ± 2.6	17.7 ± 1.5	19.3±3.6	30.6 ± 1.9	20.5 ± 1.9	33.4 ± 0.5
**12**	**Myrte EO**	8.6 ± 0.4	11.0 ± 1.7	6.8 ± 0.9	9.3 ± 0.9	10.1 ± 2.4	8.7 ± 0.6	12.8 ± 1.2	6.0 ± 0.0	11.3 ± 1.3
**13**	**Ginger EO**	6.0 ± 0.0	6.0 ± 0.0	6.0 ± 0.0	7.8 ± 2.9	6.0 ± 0.0	7.1 ± 1.3	12.4 ± 0.6	16.3 ± 1.4	11.4 ± 0.4
**14**	**Pan tropical EO**	31.1 ± 3.4	30.6 ± 2.1	23.7 ± 0.3	25.4 ± 2.1	32.0 ± 6.4	29.2 ± 1.3	61.0 ± 5.8	70.3 ± 3.4	63.0 ± 0.2
**15**	**Citral EO**	12.5 ± 1.4	10.2 ± 1.3	11.8 ± 1.6	18.4 ± 0.8	11.5 ± 1.4	10.4 ± 0.6	80.0 ± 0.0	23.0 ± 3.0	80.0 ± 0.0
**16**	**Asian EO**	8.8 ± 0.3	10.3 ± 2.6	9.2 ± 0.6	22.5 ± 1.2	9.6 ± 1.0	10.2 ± 0.7	42.7 ± 1.6	62.6 ± 6.1	80.0 ± 0.0
**17**	**Thyme thymol EO**	32.1 ± 2.2	41.4 ± 4.0	26.9 ± 3.4	31.3 ± 4.0	27.2 ± 3.7	30.5 ± 4.0	53.9 ± 2.6	38.3 ± 2.3	44.2 ± 5.3

* EO: Essential oil.

**Table 3:** Minimum inhibitory, bactericidal and fungicidal concentrations (MIC, MBC and MFC) of the selected antimicrobial compounds.

	MIC, MBC and MFC Expressed in parts-per-million, PPM (Mean Value ± SD, n =3)						
		Biosecur F440D	Pan Tropical EO	Mediterranean EO	Asian EO	Citrus EO	Tween 80 2.5%
*L. monocytogenes*	MIC	171 ± 5	621 ± 3	619 ± 2	4974 ± 0	4974 ± 0	> 12500
	MBC	171 ± 0	1241 ± 5	1237 ± 3	4974 ± 0	4974 ± 0	-
*B. subtilis*	MIC	33 ± 1	1241 ± 6	1237 ± 3	2487 ± 0	4974 ± 0	> 12500
	MBC	33 ± 0	1241 ± 0	2470 ± 0	4974 ± 0	4974 ± 0	-
*E. faecium*	MIC	142 ± 33	1241 ± 6	2474 ± 7	4979 ± 0	4974 ± 0	> 12500
	MBC	142 ± 28	2488 ± 8	4947 ± 10	4979 ± 7	4974 ± 0	-
*S. aureus*	MIC	17 ± 0	1050 ± 0	1049 ± 1	1056 ± 0	2474 ± 0	> 12500
	MBC	17 ± 0	2227 ± 0	2224 ± 0	2239 ± 0	2474 ± 0	-
*S*. Typhimurium	MIC	171 ± 5	621 ± 3	309 ± 1	1245 ± 2	4974 ± 0	> 12500
	MBC	171 ± 4	621 ± 2	619 ± 1	1245 ± 1	4974 ± 0	-
*E. coli*	MIC	114 ± 3	621 ± 3	619 ± 2	1245 ± 2	2474 ± 0	> 12500
	MBC	114 ± 2	1243 ± 5	619 ± 1	1245 ± 0	2474 ± 0	-
*C. albicans*	MIC	427 ± 12	155 ± 1	155 ± 0	311 ± 0	1245 ± 0	> 12500
	MFC	628 ± 0	621 ± 3	618 ± 1	311 ± 0	1245 ± 0	-
*A. flavus*	MIC	836 ± 23	621 ± 3	2474 ± 7	4979 ± 0	4979 ± 0	> 12500
	MFC	1261 ± 26	621 ± 1	4958 ± 5	4979 ± 7	4979 ± 0	-
*P. chrysogenum*	MIC	552 ± 11	155 ± 1	1237 ± 3	622 ± 1	1245 ± 0	> 12500
	MFC	609 ± 76	155 ± 1	2477 ± 5	622 ± 0	1245 ± 0	-

**Table 4:** Total phenols and total flavonoids content of the antimicrobial extracts.

Natural Antimicrobial Products	Total Phenols (mg gallic acid/g of AM) *	Total Flavonoids (mg catechin/g of AM) *
**Biosecur F440D**	4.38 ± 0.16 ^a^	1.26 ± 0.06 ^a^
**Pan tropical EO**	34.62 ± 3.68 ^b^	17.63 ± 1.40 ^b^
**Mediterranean EO**	220.57 ± 17.67 ^c^	34.75 ± 2.4 ^c^
**Asian EO**	1.41 ± 0.18 ^a^	0.56 ± 0.07 ^a^
**Citrus EO**	1.51 ± 0.03 ^a^	0.06 ± 0.03 ^a^

* Within each column, means with the same letter are not significantly different (*p* > 0.05); AM: Antimicrobial.

**Table 5:** Ferric reducing antioxidant power (FRAP) and Radical Scavenging Activity of the antimicrobial compounds.

Natural Antimicrobial Products	FRAP *	Radical Scavenging Activity *	
	Eq µM of Ascorbic acid/g of AM	mM Trolox	mM AA
**Biosecur F440D**	0.30 ± 0.04 ^ab^	0.28 ± 0.05 ^d^	0.29 ± 0.05 ^d^
**Pan tropical EO**	0.43 ± 0.02 ^b^	0.15 ± 0.02 ^c^	0.15 ± 0.02 ^c^
**Mediterranean EO**	0.76 ± 0.03 ^c^	0.18 ± 0.03 ^c^	0.19 ± 0.03 ^c^
**Asian EO**	0.04 ± 0.00 ^a^	0.02 ± 0.00 ^a^	0.02 ± 0.00 ^a^
**Citrus EO**	0.03 ± 0.00 ^a^	0.07 ± 0.01 ^b^	0.07 ± 0.01 ^b^

* Within each column, means with the same letter are not significantly different (*p* > 0.05).

**Table 6:** Growth rate of total mesophilic flora (TMF) in refrigerated pre-cut carrots.

Sample	Growth Rate of TMF (Ln CFU/g/day)
**Control**	0.2193
**Tsunami**	0.1852
**Antimicrobial formulation**	0.1291
